# Supplementary material for: Analysis of PPARγ Signaling Activity in Psoriasis
Source: Int J Mol Sci. 2021 Aug 10;22(16):8603. doi: 10.3390/ijms22168603 (PMC8395241; doi:10.3390/ijms22168603)
Supplement: Supplementary file 1 [file ijms-22-08603-s001.zip › Supplemental materials_Analysis of PPARg signaling activity in psoriasis/Pathway models/Models images and html files/PPARG inhibits phenotypes assotiated with psoriasis/1264386.html]

Th17 cell polarity


# Cell Process Th17 cell polarity

|  |  |
| --- | --- |
| URN | urn:agi-cellproc:prophash-5991cdc58c63903a69f077201e4afbe1 |
| Total Entities | 0 |
| Connectivity | 200 |
| Name | Th17 cell polarity |

---

|  |  |
| --- | --- |
| Pathway | PPARG inhibits phenotypes assotiated with psoriasis |
|  | efects selected |

---

|  |  |
| --- | --- |
| CellType | Th17 cell {CellType urn:agi-ncimcelltype:C2936411} |
|  | Th17 cell |

---

|  |  |
| --- | --- |
| CellProcess | cell polarity {CellProcess urn:agi-cellproc:cell%20polarity} |

---
